# Insulin stimulates β-alanine uptake in skeletal muscle cells in vitro

**DOI:** 10.1007/s00726-021-03090-9

**Published:** 2021-10-21

**Authors:** Lívia Santos, L. S. Gonçalves, Shirin Bagheri-Hanei, Gabriella Berwig Möller, Craig Sale, Ruth M. James, Guilherme Giannini Artioli

**Affiliations:** 1 Musculoskeletal Physiology Research Group, Sport, Health and Performance Enhancement Research Centre, Nottingham Trent University, Nottingham, UK; 2 Applied Physiology and Nutrition Research GroupRheumatology DivisionFaculdade de Medicina FMUSPEscola de Educação Física E Esporte, Universidade de São Paulo, São Paulo, SP Brazil; 3 College of Engineering and Physical Science, Aston University, Birmingham, B4 7ET UK; 4 Department of Life Sciences, Manchester Metropolitan University, John Dalton Building, Manchester, M1 5GD UK

**Keywords:** β-alanine, Insulin, Taurine transporter, Carnosine

## Abstract

We evaluated whether insulin could stimulate β-alanine uptake by skeletal muscle cells in vitro. Mouse myoblasts (C2C12) (*n* = 3 wells per condition) were cultured with β-alanine (350 or 700 µmol·L^−1^), with insulin (100 µU·mL^−1^) either added to the media or not. Insulin stimulated the β-alanine uptake at the lower (350 µmol·L^−1^) but not higher (700 µmol·L^−1^) β-alanine concentration in culture medium, indicating that transporter saturation might blunt the stimulatory effects of insulin.

## Introduction

Carnosine (β-alanyl-L-histidine) is found in the skeletal muscles of mammals (~ 10–40 mmol·kg^−1^ of dry muscle) (Harris et al. 2006), where it assists with pH regulation and antioxidant defences (Abe 2000; Carvalho et al. 2018). β-alanine is a constituent amino acid of carnosine and is found in low concentrations in skeletal muscle (~ 2 pmol/µL) (Goncalves et al. 2020); its low availability is rate-limiting for carnosine synthesis (Harris et al. 2006). β-alanine availability can be increased via meat consumption or dietary supplementation. Although β-alanine supplementation increases skeletal muscle carnosine (Baguet et al. 2009), only ~ 6% of the total dose ingested contributes to this (Blancquaert et al. 2016). Because muscle carnosine is associated with improved exercise capacity (Saunders et al. 2017) and has potential therapeutic benefits (Artioli et al. 2019), there is interest in developing strategies to optimise β-alanine transport into muscle cells to increase availability and enhance carnosine synthesis (Stegen et al. 2013). 

The ingestion of β-alanine with meals increased intramuscular carnosine accretion compared with the ingestion of β-alanine between meals (Stegen et al. (2013); this was attributed to a putative increase in β-alanine transporter activity due to hyperinsulinemia. In contrast, our group showed that hyperinsulinemia did not, however, increase β-alanine uptake into human skeletal muscle when β-alanine and insulin concentrations were controlled (Goncalves et al. 2020). Two transporters (TauT, PAT1) are involved in β-alanine uptake and insulin could reduce their *K*_m_ or increase their *V*_max_, an effect secondary to the stimulatory effect of insulin on Na^+^/K^+^/ATPase pump activity and, ultimately, Na^+^ influx (Clausen 2003; Sweeney and Klip 1998). Here, we sought to test the hypothesis that insulin can stimulate β-alanine uptake in skeletal muscle cells in vitro.

## Materials and methods

Mouse myoblasts (C2C12, ATCC) were cultured under standard conditions in a humidified incubator at 37 °C and 5% CO_2_ in high glucose (4500 mg·L^−1^) basal medium Dulbecco’s Modified Eagle’s Medium (DMEM) supplemented with 10% heat inactivated fetal bovine serum and 100 U·mL^−1^ penicillin/streptomycin until confluent. The medium was switched to differentiation medium (DM; high glucose DMEM supplemented with 2% horse serum and 100 U·mL^−1^ penicillin/streptomycin) and cells were allowed to differentiate for 6 days. β-alanine was dissolved in DM to a concentration of 350 or 700 µmol·L^−1^. Cell culture medium and supplements were purchased from Sigma-Aldrich. In one set of experiments, insulin was added to the DM to a concentration of 100 µU·mL^−1^, while in another set, no insulin was added. To confirm whether the effect of insulin was mediated by TauT, mouse myoblasts were differentiated as described above and treated with 1 mol·L^−1^ hypotaurine (a competitive TauT inhibitor) for 24 h in DM to inhibit β-alanine uptake. The hypotaurine was discarded, the cells were washed with PBS and incubated with a 350 or 700 µmol·L^−1^ of β-alanine with or without insulin (100 µU·mL^−1^) in DM. Cells were incubated in these formulations for 24 h. The concentration of 350 µmol·L^−1^ of β-alanine was chosen to mimic typical plasma concentrations observed following the ingestion of commonly used β-alanine doses; the concentration of 700 µmol·L^−1^ was chosen to saturate TauT (*K*_m_ ~ 40 µmol·L^−1^; Bakardjiev and Bauer 1994) and to mimic plasma concentrations elicited by high doses of β-alanine (Harris et al. 2006). The insulin concentration was chosen to mimic plasma insulin peaks following ingestion of a carbohydrate-rich meal (Stegen et al. 2013). Three independent experiments per condition, each in triplicate were carried out.

After completion, culture medium was collected, and cells washed with PBS and lysed with 2 mL of 0.5% Triton X-100 in 0.2 M NaCl. One-hundred microliters of a 5% 5-sulfosalicylic acid solution containing 500 µmol·L^−1^ of norleucine (internal standard) was added and lysate homogenised. After incubating for 30 min at 4 °C, the lysate was centrifuged at 10,000 rpm for 5 min at 4 °C, and the supernatant collected and filtered through a 0.22 µm centrifugal filter tube. Intracellular β-alanine concentrations were determined via high-performance liquid chromatography ion exchange by injecting 40 µL of the filtered supernatant through an automated amino acid analyser (Biochrom 30 + , Biochrom, Cambourne, UK). Serial dilution of amino acid standards (Merck, UK) with a constant internal standard concentration of 500 µmol·L^−1^ allowed for quantitation. The column was maintained at a 50 °C, and fluoraldehyde o-phthaldialdehyde was used for post-column derivatisation; the fluorescence detector was set at an excitation wavelength of 340 nM and an emission wavelength of 450 nM. The software EZ Chrom Elite was used to determine peak area.

### Statistical analysis

Data are presented as mean ± SD with 95% confidence intervals (95% CI). β-alanine concentrations in cell lysates were compared between conditions (supplemented with β-alanine with or without hypotaurine and insulin, and non-supplemented controls) with one-way ANOVA followed by the Tukey *post-hoc* where appropriate. Data were analysed in two data sets, those supplemented with 350 µmol·L^−1^ or those with 700 µmol·L^−1^ of β-alanine. Alpha level was set at 5%. All analyses were carried out in the SAS statistical software (v.9.3; SAS Institute, Cary, NC).

## Results

### *Cells supplemented with 350 µmol·L*^*−1*^* of β-alanine (Fig. **1**)*

**Fig. 1:**
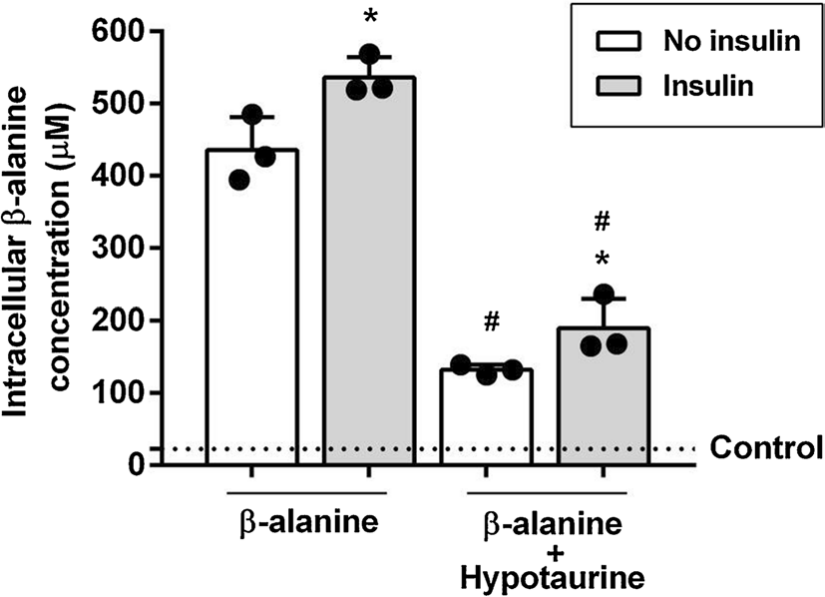
Intracellular β-alanine concentration determined in lysate of myoblasts treated for 24 h with DM supplemented with 350 μmol·L^−1^ β-alanine with or without insulin (100 µU·mL^−1^) after treatment with hypotaurine or no treatment. Mean values measured in control cells are depicted by dotted lines. One-way ANOVA: all conditions are significantly different from control (all *p* < 0.0001). Tukey *post-hoc*: no insulin *vs*. insulin: *p = 0.002; no insulin *vs.* no insulin + hypotaurine ^#^*p* < 0.001; insulin *vs.* insulin + hypotaurine ^#^*p* < 0.001; no insulin + hypotaurine *vs*. insulin + hypotaurine: *p = 0.04. *n* = 3 per condition

Incubation with 350 µmol·L^−1^ of β-alanine increased β-alanine concentrations in cell lysates in all conditions (*F* = 339.13; *p* < 0.0001). Insulin further increased intracellular β-alanine accrual in comparison with cells not treated with insulin (*t* = − 6.12; *p* = 0.002; 95% CI − 157.96 to − 43.93). Treatment with hypotaurine resulted in lower β-alanine concentrations in cell lysates in comparison with cells not treated with hypotaurine; this was shown either with (*t* = 21.01; *p* < 0.0001; 95% CI 289.77–403.80) or without insulin (*t* = 18.37; *p* < 0.0001; 95% CI 246.17–360.20). Despite the inhibitory effect of hypotaurine on β-alanine uptake, insulin increased β-alanine in hypotaurine-treated cells (*t* = − 3.48; *p* = 0.04; 95% CI − 114.37 to − 0.34, vs. hypotaurine-treated without insulin).

### *Cells supplemented with 700 µmol·L*^*−1*^* of β-alanine (Fig. **2**)*

**Fig. 2:**
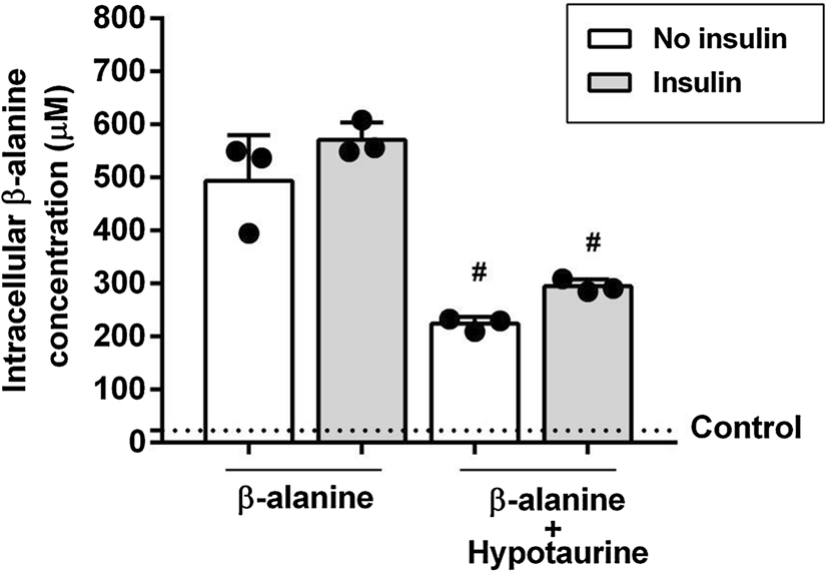
Intracellular β-alanine concentration determined in lysates of myoblasts treated for 24 h with DM supplemented with 700 μmol·L^−1^ β-alanine with or without insulin (100 µU·mL^−1^) after treatment with hypotaurine or no treatment. Mean values measured in control samples are depicted by dotted lines. One-way ANOVA: all conditions are significantly different from control (all *p* < 0.0001). Tukey *post-hoc:* no insulin *vs.* no insulin + hypotaurine ^#^*p* = 0.0001; insulin *vs.* insulin + hypotaurine ^#^*p* = 0.0001. *n* = 3 per condition

Incubation with 700 µmol·L^−1^ of β-alanine led to an increase in β-alanine concentration measured in cell lysate in all conditions compared to control (*F* = 104.68; all *p* < 0.0001). No significant differences were shown when comparing β-alanine between cells treated with and without insulin (*t* = − 2.56; *p* = 0.17; 95% CI − 181.77 to 27.18). Treatment with hypotaurine resulted in lower β-alanine concentrations in cell lysates compared with cells not treated with hypotaurine; this was shown with (*t* = 9.13; *p* = 0.0001; 95% CI 171.75–380.71) and without insulin (*t* = 8.92; *p* = 0.0001; 95% CI 165.19–374.14). Insulin did not increase β-alanine concentration in cells treated with hypotaurine (t = − 2.34; *p* = 0.23; 95% CI − 175.20 to 33.75).

## Discussion

β-alanine is primarily taken up into skeletal muscle cells in a saturable process undertaken by TauT, a Na^+^ and Cl^−^ dependent transmembrane transporter driven by Na^+^ flux, which is secondary to the action of the Na^+^/K^+^-ATPase pump (Jessen 1994). Since the Na^+^/K^+^/ATPase pump and Na^+^ influx are stimulated by insulin (Clausen 2003), hyperinsulinemia could increase TauT efficiency (Stegen et al. 2013), although direct experimental evidence for this is lacking. Herein, we showed that insulin can stimulate β-alanine transport into skeletal muscle cells, but only under lower (350 µmol·L^−1^) and not higher (700 µmol·L^−1^) β-alanine concentrations in culture medium, indicating that transporter saturation might blunt the stimulatory effects of insulin. The reduced β-alanine uptake after incubation with hypotaurine confirmed that β-alanine transport to skeletal muscle cells is, at least in part, mediated by TauT, although we acknowledge that hypotaurine might impact other pathways that were not account for in this investigation.

Stegen et al. (2013) showed that chronic β-alanine supplementation in humans increased muscle carnosine concentration in the *m. soleus*, but not *m. gastrocnemius*, when β-alanine was ingested with meals (high insulin) when compared to between meals (low insulin). Conversely, an in vivo human study, using the hyperinsulinemic–euglycemic clamp, showed that hyperinsulinemia did not increase β-alanine uptake when substrate concentrations exceeded the *V*_max_ of TauT nor when it was below saturation of the β-alanine transporters (Goncalves et al. 2020). To further investigate the potential for insulin to influence β-alanine uptake by skeletal muscle, we used a cell culture model better suited to specifically test this hypothesis. Under saturating conditions of β-alanine (700 μmol·L^−1^), no stimulatory effect of insulin on β-alanine uptake was shown, corroborating our earlier findings in humans (Goncalves et al. 2020) and supporting the notion that insulin does not increase the *V*_max_ of TauT. In contrast, when myoblasts media were supplemented with 350 μmol·L^−1^ of β-alanine, insulin induced greater β-alanine uptake, both under normal conditions and when the TauT competitive inhibitor hypotaurine was administered. These findings indicate that, under lower β-alanine concentrations, insulin potentiates β-alanine transport into skeletal muscle, most likely by reducing the *K*_m_ of TauT, resulting in its increased activity and higher affinity for its substrates (Richter et al. 2019). We speculate that this is mediated by increased Na^+^ gradients, secondary to increased Na^+^/K^+^/ATPase pump activity.

Although TauT is a major β-alanine transporter into muscle cells (Jessen 1994), a notion that is supported by the dramatic decrease in β-alanine accrual in the hypotaurine-treated cells, β-alanine can also be transported by PAT1, a Na^+^-independent, H^+^-dependent transporter (Frolund et al. 2010). Insulin could act upon PAT1 by stimulating the Na^+^/H^+^ exchanger (Klisic et al. 2002) and, thus, one could argue that the higher β-alanine uptake with 350 μmol·L^−1^ of β-alanine could also have occurred via a reduced *K*_*m*_ of the PAT1 transporter. However, it is known that Na^+^ increases PAT1 activity only in acidic conditions (pH ~ 5.5–6.0) (Chen et al. 2003; Daniel et al. 1991), and since our experiments were performed within the pH range of resting skeletal muscle (pH ~ 7.0), an effect of insulin on the Na^+^/H^+^ exchanger is unlikely. Assuming this to be correct, there would be little effect on PAT1 activity, meaning that the stimulatory effect of insulin would be due to the increased affinity of TauT for β-alanine.

In conclusion, we demonstrate that insulin stimulates β-alanine uptake in skeletal muscle cells in vitro, possibly due to the increased affinity of TauT for β-alanine, but only when substrate concentration does not exceed its *V*_max_. More research is needed to determine whether this effect has relevant implications for whole-body physiology.
